# SALMO and S_3_M: A Saliva Model and a Single Saliva Salt Model for Equilibrium Studies

**DOI:** 10.1155/2015/267985

**Published:** 2015-02-04

**Authors:** Francesco Crea, Concetta De Stefano, Demetrio Milea, Alberto Pettignano, Silvio Sammartano

**Affiliations:** ^1^Dipartimento di Scienze Chimiche, Università di Messina, Viale F. Stagno d'Alcontres 31, 98166 Messina, Italy; ^2^Dipartimento di Fisica e Chimica, Università di Palermo, Viale delle Scienze, Ed. 17, 90128 Palermo, Italy

## Abstract

A model of synthetic saliva (SALMO, SALiva MOdel) is proposed for its use as standard medium in *in vitro* equilibrium and speciation studies of real saliva. The concentrations come out from the literature analysis of the composition of both real saliva and synthetic saliva. The chief interactions of main inorganic components of saliva, as well as urea and amino acids, are taken into account on the basis of a complex formation model, which also considers the dependence of the stability constants of these species on ionic strength and temperature. These last features allow the modelling of the speciation of saliva in different physiological conditions deriving from processes like dilution, pH, and temperature changes. To simplify equilibrium calculations, a plain approach is also proposed, in order to take into account all the interactions among the major components of saliva, by considering the inorganic components of saliva as a single 1 : 1 salt (MX), whose concentration is *c*_MX_ = (1/2)∑*c*_*i*_ (*c*_*i*_ = analytical concentration of all the ions) and *z* ion charge calculated as z=±(*I*/*c*_MX_)^1/2^ = ±1.163. The use of the Single Saliva Salt Model (S_3_M) considerably reduces the complexity of the systems to be investigated. In fact, only four species deriving from internal ionic medium interactions must be considered.

## 1. Introduction

Chemical speciation studies in real systems are usually very complex, due to the wide number of interactions that must be taken into account, which lead to the formation of several species of different stability [1–7]. This is particularly true in the case of biological fluids, where not only the composition varies from fluid to fluid, but it may also depend on several other factors like,* for example*, different physiological conditions, age, kind of living organism, and diseases [8, 9]. These changes are usually mainly responsible of the differences between results obtained and predictions made by* in vitro* and/or* in silico* studies and what is effectively observed* in vivo* [9–13]. That is why, during the years, several “artificial media” have been proposed to simulate the composition of a wide number of real systems (with particular reference to biological fluids), with the aim of performing various studies in conditions that are as close as possible to those effectively found in the reality: typical is the use of artificial seawaters in environmental studies (*e.g.*, [14, 15] and references therein) or simulated body fluids in the pharmaceutical field (*e.g.*, [9] and references therein). Unfortunately, the simple preparation and use of an artificial medium is not sufficient when performing rigorous chemical speciation studies. This is due to the fact that the investigation of the “distribution of an element amongst defined chemical species in a system” (*i.e.*, its speciation [16]) is based on the evaluation of the main interactions of this element with all other components in the system and on determination of the stability of species formed, but this process requires the preliminary knowledge of all the interactions occurring between all components already present in the system. In other words, a chemical speciation model of the biological fluid itself is necessary prior to any investigation on the speciation of any other component in that fluid. Furthermore, assuming that a speciation model of the fluid is available, the above-cited variability of conditions makes also the assessment of their effect on the speciation necessary: the dependence of the stability and the distribution of various species on chemical (*e.g.*, kind and concentration of components, ionic strength, and pH) and/or physical (*e.g.*, temperature) parameters must be known to build accurate speciation models.

During the years, this group has been involved also in this kind of work, proposing the use of new synthetic media (like,* e.g.*, a synthetic seawater [14]), providing chemical speciation models of natural waters (*e.g.*, seawater [15]) and biological fluids (*e.g.*, urine [17] and blood plasma [18]), as well as alternative approaches to the study of chemical equilibria in these media [19].

In this contribution, a model of synthetic saliva (SALMO, SALiva MOdel) is proposed for its use as standard medium in* in vitro* equilibrium and speciation studies of real saliva. In fact, though various artificial media simulating saliva have been proposed since many years and are still used in several fields (see,* e.g.*, [9, 20–22] and references therein), to our knowledge no “reference” speciation models are available in literature, hampering the use of these media in chemical speciation studies.

Based on an approach previously adopted for synthetic seawater [19] and successfully tested in several speciation studies (*e.g.*, [23–30]), a simpler model is also proposed, to simplify equilibrium calculations, by considering the inorganic components of saliva as a single 1 : 1 salt (MX), reducing the complexity of the systems to be investigated.

## 2. Synthetic Saliva Composition and Formulation

As well known, real saliva has a very complex and variable composition, depending on several factors, so that its exact replication is almost impossible ([8–10, 20–22, 31–33]). Nevertheless, from the point of view of chemical speciation studies, it is initially possible to neglect many constituents of lower interest (in this case!), such as,* for example*, proteins, enzymes, bacteria, and cellular material. In fact, any speciation study in this medium should start from the interactions of the element or compound under investigation with the main inorganic components of saliva and, successively, extending it to some organic ligands. Bearing this in mind, we analysed the most relevant literature findings on the composition of real and artificial saliva from present time (November 2014) to years 1983 and 2001 ([8, 9, 20–22, 32–34] and references therein), when Lentner (in the Geigy Scientific Tables [8]) and Gal and coworkers [20] published two updated, comprehensive, and detailed revisions of previous contributions on the composition of real and artificial saliva, respectively. Geigy tables [8] represent a “standard” and well considered reference in the medical and biological field about the composition of a lot of biological fluids, including saliva. They report data about the composition of hundreds of saliva samples, including stimulated and not stimulated and organic and inorganic components and differences of sex, age, and smoking: it is a very comprehensive reference reporting several chemicophysical parameters. Analogously, the work by Gal et al. [20] is one of the most successful and quite accurate attempts of building synthetic saliva. Also in this case, a wide number of synthetic (about 60) and natural saliva compositions are taken into account and critically evaluated. On the basis of data reported in the above-cited literature ([8, 9, 20–22, 32–34] and references therein), we here propose a saliva model (SALMO), which is able to summarize the main interactions of main inorganic components of saliva, as well as urea and amino acids. Its composition is reported in Table 1. During model development, higher weights have been given to data related to stimulated saliva, since this situation is probably the most important in many cases when speciation studies are required (stimulated saliva is produced,* e.g.*, during oral drug absorption [12], eating, and drinking). The given composition takes into account (with different weights) both unstimulated saliva and stimulated (from different origin) saliva. The synthetic saliva according to SALMO can be prepared as reported in Table 2. As representative of amino acids, glycine can be used. Worthy of a mention is also the fact that, considering usual pH values of saliva, carbonate and phosphate ligands have been considered in Tables 1 and 2 as hydrogen carbonate and hydrogen phosphate, respectively, and must be added in this form in the formulation.

## 3. Speciation Model

### 3.1. Data Sources

In a formulation like the one already proposed, containing thirteen components (fourteen if one also considers H^+^/OH^−^), it is immediately evident that the number of species that could be formed is consistent. The stability constants to be taken into account refer to protonation equilibria of the ligands, hydrolysis of cations, all possible species between cations and anions (including weak complexes), amino acid species with both cations and anions (due to the presence of both aminic and carboxylic groups), and urea interactions. Moreover, it is also well known that, in multicomponent solutions, the formation of mixed (ternary or higher) species is possible and usually favoured [4, 35], so that these species cannot be neglected in a correct speciation model. On this basis, a huge dataset of stability constants is necessary to build the model and, furthermore, they must be available at the effective ionic strength and temperature of the system under study. In this work, the most of these data have been taken from the most common general stability constant databases [36–41] and, when possible, from some reviews and/or papers dedicated to specific ligands and/or cations, by this and other groups (*e.g.*, [17, 42] for glycine, [43–46] for phosphate, [47] for thiocyanate, [48, 49] for fluoride, [50] for carbonate, [51, 52] for urea, and for [43, 53, 54] sulphate; all considering references therein). Though the most of last references were already taken into account in the above-cited databases, they have been equally consulted because they contain some more specific information like, for example, the parameters for modelling the dependence of the stability constants of various species on ionic strength and/or temperature.

### 3.2. Expression of Results

All hydrolysis, protonation, and complex formation constants reported in the paper are given according to the overall equilibrium:

(1) pMm+p′M′m′+qLl+q′Ll′+rH+ =MpM′p′LqL′q′Hr(pm+p′m′+lq+l′q′+r)βpp′qq′r,

where the superscripts “*m*” and “*l*” denote the charges of cations and ligands, with their corresponding signs. The extra cations (M′) and ligands (L′) were taken into account in the general equilibrium to refer only to the formation of mixed species: in all other cases, *p*′ = *q*′ = 0. For simple species, when *p* = 0, (1) refers to the ligand protonation constants; negative *r* index refers to the formation of hydroxo-complexes and, in particular, to the cation hydrolysis constants when also *q* = 0. If not necessary, the charges of the various species are omitted for simplicity.

If not differently specified, errors are expressed as ± standard deviation, and formation constants, concentrations, and ionic strengths are expressed in the molar concentration scale (*c*, mol L^−1^). Rigorously, this scale is temperature dependent and should not be used to express quantities at different temperatures. In those cases, temperature independent concentration scales, such as the molal scale (*m*, mol (kg solvent)^−1^) should be preferred. Nevertheless, the molar scale is more frequent and “practical” and, in relatively small temperature ranges and ionic strength values, errors associated to its use of the molar scale on behalf of the molal scale may be negligible [55]. A detailed description of errors associated to data reported in this paper and to their reliability is given in next sections.

### 3.3. The SALMO Model: Main and Minor Species

According to the data sources described in previous paragraph, the speciation of SALMO is given by 93 species, listed in Table 3 together with the corresponding stability constants at *t* = 37°C and *I* = 0.15 mol L^−1^. Due to the availability of many data at these temperature and ionic strength values (because they approach many physiological conditions like,* e.g.*, blood plasma [8]) they have been taken as reference. The same table also reports the parameters for the dependence of the stability constants on ionic strength and temperature, though this aspect will be discussed in next paragraphs.

Looking at the species (and at their corresponding stability constants) reported in Table 3, a series of comments and clarifications is necessary. Of the 93 species reported, some (those we call the “main species” like, e.g., many protonation constants or some alkaline earth complexes) are more important than others (the “minor species”) and better characterized (*i.e.*, many stability constants, as well as other thermodynamic parameters, are reported in literature in different conditions). In contrast, many “minor species” have been less investigated or, in some worse cases, never reported, though it is reasonable that they may be formed in systems as complex as these. We refer, for example, to the formation of some mixed MM′LH_*r*_ or MLL′H_*r*_ species.

In fact, according to Beck and Nagypàl [35], in a ternary system (A, B, C), if A forms binary complexes with both B and C (i.e., AB_2_ and AC_2_), the formation of the ABC species is possible and statistically favored, since the probabilities of formation of AB_2_, AC_2_, and ABC are 0.25, 0.25, and 0.5, respectively. Briefly, for the generic equilibrium

(2) $$\begin{array}{c} p{\text{AB}}_{\left(p+q\right)}+{q\mathrm{AC}}_{\left(p+q\right)}=\left(p+q\right){\text{AB}}_{p}{\text{C}}_{q} \end{array}$$

the probability of formation of the mixed species is given by

(3) $$\begin{array}{c} X_{\text{stat}}={\left[\frac{\left(p+q\right)!}{\left(p!q!\right)}\right]}^{\left(p+q\right)}. \end{array}$$

A more accurate approach for the calculation of the statistical stability of mixed species takes into account the specificity of chemical interactions between various components [4].

In the above-described ternary system, the statistical value of the formation constant relative to equilibrium (*i.e.*, (2) with *p* = *q* = 1)

(4) $$\begin{array}{c} {\text{AB}}_{2}+{\mathrm{AC}}_{2}=2\text{ABC} \end{array}$$

can be estimated knowing the stepwise formation constants of simple species:

(5) $$\begin{array}{c} X_{\text{stat}}=2+\frac{K_{1}^{\text{A}}}{K_{1}^{\text{B}}}\sqrt{\frac{K_{2}^{\text{B}}}{K_{2}^{\text{A}\;}}}+\frac{K_{1}^{\text{B}}}{K_{1}^{\text{A}}}\sqrt{\frac{K_{2}^{\text{A}}}{K_{2}^{\text{B}\;}}}. \end{array}$$

The stability constant of a mixed species can be, therefore, either estimated statistically

(6) p+qlog⁡βABpCq=log⁡Xstat+plog⁡βABp+q+qlog⁡βAC⁡p+q

or can be experimentally determined once the stability of the corresponding simple species is known. In this case, (6) may be rearranged to

(7) log⁡Xexp⁡=p+qlog⁡βABpCq−plog⁡βABp+q−qlog⁡βAC⁡p+q.

The same approach could be also adopted for the estimation of other thermodynamic formation parameters than stability constants (*e.g.*, formation enthalpy or entropy changes) [56]. Higher log *X*_exp⁡_ values than corresponding log *X*_stat_ indicate that the formation of mixed species is thermodynamically favored and are a numerical index of the extra stability of mixed species with respect to simple ones. This extra stability has been observed for several systems, providing evidence of the formation of various mixed species (like it has been supposed in this paper), which are able to affect the speciation and the thermodynamic properties of systems where they are formed [56–62].

That is why some mixed species, determined in this way, have been reported in Table 3 and taken into account in the model (values for other mixed species were already been determined experimentally and available in literature like,* e.g.*, some glycinate [42] or phosphate [46] complexes). Their formation could be generally low, but, according to changes in saliva conditions (*e.g.*, pH, ionic strength, temperature, and presence of other substances), some of these “minor species” may be formed in nonnegligible amounts.

### 3.4. Dependence of the Stability Constants on Ionic Strength and Temperature

As already discussed, saliva conditions may vary, so that the use of the stability constant values reported in Table 3 at other temperatures and ionic strengths than the reference ones (*i.e.*, *I* = 0.15 mol L^−1^ and *t* = 37°C) may represent a further source of error in the evaluation of saliva speciation. Fortunately, these errors may be significantly reduced by the calculation of these constants at the correct ionic strength and temperature values, by applying some common and well known models and equations.

In this work, the dependence of various formation constants on ionic strength has been taken into account by an Extended Debye-Hückel (EDH) type equation:

(8) $$\begin{array}{c} \log\beta^{\prime }=\log\beta_{\text{ref}}-z^{*}\left({\text{DH}}^{\prime }-{\text{DH}}_{\text{ref}}\right)+C\left(I^{\prime }-I_{\text{ref}}\right), \end{array}$$

where *C* is an empirical parameter (reported in Table 3 for every species), and DH is the Debye-Hückel term

(9) $$\begin{array}{c} \text{DH}=-\frac{z^{*}0.51I^{1/2}}{\left(1+1.5I^{1/2}\right)} \end{array}$$

with

(10) $$\begin{array}{c} z^{*}=\sum {\left(\text{charges}\right)}_{\text{reactants}}^{2}-\sum {\left(\text{charges}\right)}_{\text{products}}^{2}, \end{array}$$

where log⁡*β*′, DH′, and *I*′ are referred to the desired ionic strength and log⁡*β*_ref_, DH_ref_, and *I*_ref_ to the reference one. Only after a new set of stability constants is obtained at a desired ionic strength, it can be recalculated at the desired temperature, by the van't Hoff equation:

(11) $$\begin{array}{c} \log\beta =\log\beta^{\prime }+a\left(\frac{1}{T_{\text{ref}}}-\frac{1}{T}\right), \end{array}$$

where *T* is the desired temperature in Kelvin (*t*°C + 273.15). As is in (11), “*a*” parameter (reported in Table 3) takes directly into account the contribution of the formation enthalpy changes, the universal gas constant, and the conversion from natural to decimal logarithms. Parameters reported in Table 3 are generally valid at *I* ≤ 0.5 mol L^−1^ and in the temperature range 25 ≤ *t*/°C ≤ 40. By using (11), the SALMO stability constant datasets at the *I*_ref_ ionic strength were calculated at four different temperatures and are shown in Table 4.

### 3.5. The Speciation of Saliva according to SALMO

The huge number of species reported in Table 3 (and Table 4) is a clear indication of the complex network of interactions occurring between different saliva components. As direct consequence of these interactions, the free concentration of saliva components is never equivalent to the analytical (total). SALMO, designed to be employed during speciation studies in saliva, can also be used for the calculation of the free concentrations of different components of saliva of given composition. For example, considering the analytical concentrations of components of the synthetic saliva reported in Table 1, the free concentration of its components at two temperatures and two pH values has been calculated by SALMO (using common speciation programs [63, 64]). These results are summarized in Table 5 and demonstrate what was already stated: all the internal ionic interactions between the saliva components cannot be neglected because they lower the concentration of free ions. For example, at *t* = 37°C, more than 40% of Mg^2+^ and Ca^2+^ are complexed, while urea exists almost entirely as free form. Worth mentioning is also the fact that, instead of giving free phosphate and carbonate concentrations, we preferred to report their monoprotonated species as reference, since they are more relevant for the speciation of saliva and other natural and biological fluids [8].

## 4. The Single Saliva Salt Model, S_3_M

All considerations just presented on the advantages of using a synthetic medium cannot lead the reader astray from the fact that the speciation model proposed, as usually occurs for many other models of multicomponent systems, is “quite complex.” Performing the speciation study of an “external” component in this (or other) medium would result in the evaluation of all its relevant interactions with all saliva components, with the possibility of forming many species, whose stability constants should be determined and then added to the model. As a consequence, if we take into account these interactions when SALMO is used in the speciation studies of saliva, along with the other species formed by other components, a considerable number of species need to be considered.

To bypass this problem, in order to simplify equilibrium calculations, a simpler approach is proposed here, based on the Single Salt Approximation adopted for synthetic seawater [19] and successfully tested in several speciation studies (*e.g.*, [23–30]). In order to take into account all the interactions among the major components of saliva, we considered the inorganic components of saliva given in Table 1. (*i.e.*, all components except amino acids and urea) as a single 1 : 1 salt (MX), whose concentration is

(12) $$\begin{array}{c} c_{\text{MX}}=\frac{1}{2}\sum c_{i} \end{array}$$

(*c*_*i*_ = analytical concentration of all the ions) and with an ion charge (*z*) calculated as

(13) $$\begin{array}{c} z=\pm {\left(\frac{I}{C_{\text{MX}}}\right)}^{1/2}=\pm 1.163. \end{array}$$

Main characteristics of the Single Saliva Salt (MX) are summarized in Table 6.

The use of the Single Saliva Salt allowed us to build a much simpler but equally reliable speciation model for synthetic saliva than SALMO. In fact, the Single Saliva Salt Model (S_3_M) considerably reduces the complexity of the systems to be investigated, since only four species deriving from internal ionic medium interactions must be considered. These species represent the self-association of the salt, the hydrolysis of the cation M, and the protonation and the deprotonation of the anion X (coherently with the fact that HPO_4_^2−^ and HCO_3_^−^ were used as reference components and that they may be deprotonated). Overall stability constants relative to the formation of the species of S_3_M are reported in Table 7 at the reference ionic strength and temperature, together with their dependence parameter (according to what has been done for SALMO). Further details on the procedure adopted to calculate these parameters may be found, for example, in [19].

**Figure 1:**
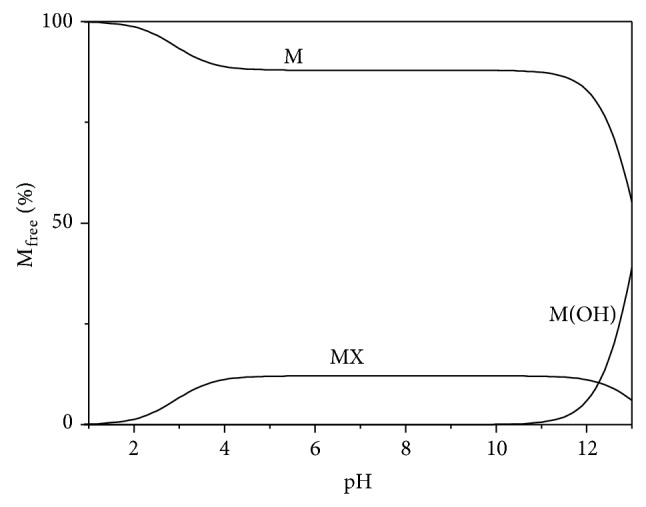
Distribution diagram of species of cation “M” of synthetic saliva* versus* pH, at *t* = 37°C, according to S_3_M.

**Figure 2:**
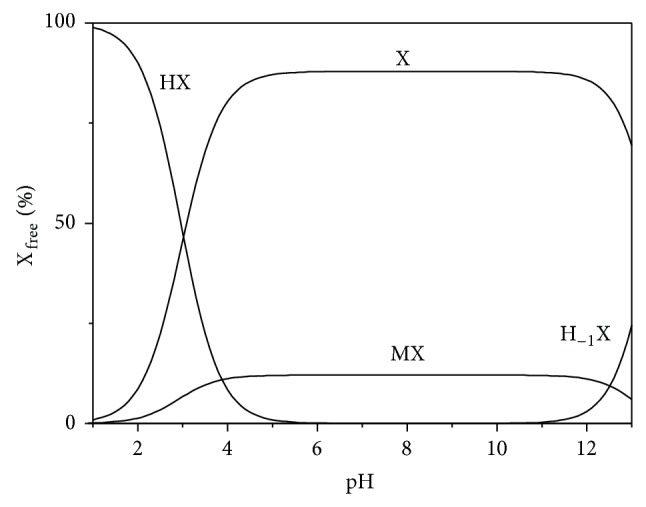
Distribution diagram of species of anion “X” of synthetic saliva* versus* pH, at *t* = 37°C, according to S_3_M.

By means of S_3_M, all the internal interactions between the inorganic components of synthetic saliva are taken into account considering just four equilibria. As a consequence, the speciation of “external” components in saliva can be studied just by considering its interactions with the “M” and “X” ions of saliva (reducing the complexity to “just” a ternary one metal + one ligand + one component system). The importance of various MX species, according to S_3_M, is better realized looking at Figures 1 and 2, where two speciation diagrams are reported for M^1.163+^ and X^1.163−^ species, respectively. As can be noted, in the pH range 3 ≤ pH ≤ 9, the M(OH) and H_−1_X species can be neglected. In the pH range of interest, ~12% of the MX salt is self-associated, whilst the rest is present as free X and M. Only below pH ~ 5 the protonation of the ligand becomes significant.

## 5. The Reliability of the Models 

Both SALMO and S_3_M, as well as the synthetic saliva composition proposed, are “models.” Models are built to describe and/or interpret some observed phenomena, but, for their intrinsic nature, they are “approximations”: a “good model” should be a good compromise between simplicity of use and reliability of results obtained. Also in the case of models proposed here, some aspects must be discussed more in detail.

### 5.1. Purposes of the Models

We already discussed about the composition and the formulation of the synthetic saliva proposed. As already stated, several other compounds could have been included in the formulation, other concentrations could have been used, or some other modifications could have been possible. As we intended, this formulation would represent the “starting point” for specific studies,* that is*, those addressed at understanding the thermodynamic behavior and the speciation of components “of” and “in” the saliva system. From just this point of view, more attention should be (and it has been) given to the chemical and physical aspects of saliva system (like,* e.g.*, ionic strength, temperature, and ionic composition), instead of others that are less important for the aims proposed (*e.g.*, presence of enzymes and “living material”).

A similar consideration can be done for SALMO. Its purpose is to describe the speciation of a complex system like saliva and to take into account the most relevant interactions in this medium, but what does “relevance” mean? Of course, of the 93 species reported, many could have been neglected, reducing sensibly this number (to about 60–70 species). Nevertheless, though the formation percentage of a single minor species could be “not significant,” all species globally contribute to give a comprehensive picture of what really happens in saliva. This is also the reason why some species (some mixed) never reported in literature before have been estimated in this work. Furthermore, the discussion about the possibility that these species could really be formed, as well as their stability, has already been done above.

A last consideration is necessary for S_3_M. Its peculiarity and its simplicity should not rule out the fact that this interaction model is directly derived from its parent model SALMO, maintaining all the characteristics of a comprehensive speciation model.

### 5.2. Errors Associated to the Stability Constants and Influence on “Real” Speciation

Both SALMO and S_3_M are thermodynamic models, based on stability constants and parameters for their dependence on ionic strength and temperature. As already stated, some of these values have never been determined experimentally or are present in literature at other conditions than those of interest and have been estimated by taking into account well known “facts” like, for example, (a) the similarities of the thermodynamic behaviour of similar species (*e.g.*, concerning the dependence on ionic strength and temperature, see [65, 66]) and/or (b) well defined trends in the stability of complexes of homogeneous ligand classes (see,* e.g.*, [67–70]). As a direct consequence, we associated a wide range (±0.01–0.1 standard deviation, see Table 3) to the errors of the stability constants reported in this work. This width comes out from the differences between well known stability constants and ionic strength and temperature dependence parameters (with lower standard deviations than 0.01,* e.g.*, *K*_w_, and some hydrolysis and protonation constants) and some estimated values (with higher values). Isolating this concept from the context of this work, from a pure thermodynamic point of view, errors like those reported here for a simple stability constant appear to be quite high. Nevertheless, during speciation studies, especially for very complex multicomponent systems, the critical aspect is the propagation of these errors on the “real” speciation of a given system. ES4ECI [63], the program we used to calculate the concentration of different species (as also the free components reported in Table 5) is able to propagate the errors of stability constants (included in the input) on the formation percentage of different species. As can be noted in Table 5, so (apparently) high standard deviation in the stability constants used results in an acceptable uncertainty in the formation percentage of species (below 3% for free components in Table 5). For practical uses and applications to real systems, this order of uncertainty is common and is generally accounted as “low,” supporting our assumptions of the reliability of the proposed models.

## 6. Literature Comparisons

As stated above, saliva composition is very variable. As a consequence, we already pointed out that many “different” artificial saliva models of very “different” composition have been proposed during the years, for many “different” purposes. Depending on the aim of studies performed, single components or classes of components may be included/excluded from the formulation as, for example, done by Björklund et al. [21], who considered vitamins, enzymes, and glycoproteins (mainly mucins) in the artificial saliva they prepared for studying the influence of different carbon sources on bacterial growth. To our knowledge, neither artificial media have been ever prepared, nor have complex formation models been proposed specifically for speciation studies of saliva. The closest attempt is represented, once again, by the comprehensive review by Gal et al. [20]: in that work, some chemicophysical aspects have been considered, like, for example, the buffering effect of saliva, its ionic strength, and pH, affected by the presence of selected ions (Ca^2+^, SCN^−^, HCO_3_^−^, and HPO_3_^2−^), which lead to the formation of selected species. Some acid-base titrations of saliva have also been simulated, and the free concentrations of some species have also been calculated using literature stability constants. From the comparison of data reported by Gal et al. and results obtained in this work, it is still possible to state that an excellent agreement exists, at least for the order of magnitude of free concentrations of some components (in mol L^−1^). At pH 6.8 and *t* = 22°C, Gal et al. report [HCO_3_^−^] = 5.54*E* − 03, [Cl^−^] = 2.15*E* − 02, [Ca^2+^] = 1.14*E* − 03, [NH_4_^+^] = 3.28*E* − 03, [HPO_4_^2−^] = 1.70*E* − 03, and [SCN^−^] = 1.95*E* − 03. In this work (Table 5), at pH = 6.5 and *t* = 25°C we have [HCO_3_^−^] = 7.17*E* − 03, [Cl^−^] = 2.46*E* − 02, [Ca^2+^] = 1.50*E* − 03, [NH_4_^+^] = 3.35*E* − 03, [HPO_4_^2−^] = 1.95*E* − 03, and [SCN^−^] = 1.90*E* − 03. The discrepancies can be ascribed to the differences in the saliva composition, but, mainly, in the number and species and in the stability constants considered (taken from literature at *t* = 25°C and *I* = 0 mol L^−1^). In fact, the same authors state in their work that only species where the thermodynamic constants were known were taken into account. This last consideration strengthens the necessity of a more comprehensive and dedicated speciation model for saliva.

## 7. Final Remarks

Results reported in this paper can be summarized as follows:

- formulation of synthetic saliva specifically aimed at thermodynamic and speciation studies is reported here for the first time, based on several literature findings of compositions of real and synthetic saliva in various conditions;
- comprehensive complex formation model of this saliva, based on the formation of 93 species, has been proposed for the modelling of its speciation at different ionic strength and temperatures;
- another simpler model, based on the “Single Salt Approximation”, is also proposed, in which the inorganic components of saliva are taken into account as a single 1 : 1 salt, reducing the complexity of the saliva system;
- data reported have been critically analysed in terms of reliability of results obtained and applicability to real systems.

## Figures and Tables

**Table 1:** Analytical concentrations of components in synthetic saliva.

Cation	*c*/mmol L^−1^	Anion	*c*/mmol L^−1^
Na^+^	20.3	Cl^−^	25.3
K^+^	28.9	HCO_3_^−^	11.45
Ca^2+^	2.1	HPO_4_^2−^	8.5
Mg^2+^	0.5	SCN^−^	1.95
NH_4_^+^	3.5	F^−^	0.0025
		SO_4_^2−^	1.1

∑*c*_(charge)_^(a)^	57.9		57.9
AA^±^^(b)^	0.35		
Urea	3.3		

^(a)^Concentration of charges of cations and anions; ^(b)^amino acids.

**Table 2:** SALMO composition.

Salt	*c*/mmol L^−1^
NaCl	8.8525
KCl	7.75
CaCl_2_	2.1
MgCl_2_	0.5
K_2_SO_4_	1.1
NaF	0.0025
NaHCO_3_	11.45
K_2_HPO_4_	8.5
NH_4_Cl	3.5
KSCN	1.95
Glycine^(a)^	0.35
Urea	3.3

^(a)^Taken as reference amino acid.

**Table 3:** Stability constants of SALMO species at *t=37*°C and *I=0.15* mol L^−1^ ionic strength and corresponding parameters for their dependence on ionic strength and temperature (by (8)–(11)).

ID	Species^(a)^	log⁡*β*_ref_^(b)^	*C*	*z* ^*^	*a*
1	(OH)	−13.45	−0.41	−2	2814
2	(Na)(OH)	−13.25	−0.06	0	2602
3	(K)(OH)	−13.35	−0.06	0	2337
4	(Ca)(OH)	−12.56	0.25	2	2374
5	(Mg)(OH)	−11.45	0.25	2	2178
6	(NH_4_)(OH)	−8.90	−0.06	0	2745
7	(SCN)(H)	−1.29	0.36	2	2862
8	(Na)(SCN)	−0.51	0.36	2	53
9	(K)(SCN)	−0.49	0.36	2	53
10	(Mg)(SCN)	−1.19	0.66	4	53
11	(Ca)(SCN)	−1.19	0.66	4	53
12	(NH_4_)(SCN)	−0.51	0.36	2	53
13	(F)(H)	3.05	0.36	2	705
14	(F)_2_(H)	3.55	0.41	2	217
15	(Na)(F)	−0.51	0.36	2	636
16	(K)(F)	−0.49	0.36	2	636
17	(Mg)(F)	1.37	0.66	4	689
18	(Ca)(F)	0.77	0.66	4	742
19	(NH_4_)(F)	−0.51	0.36	2	636
20	(Na)(Cl)	−0.51	0.36	2	−424
21	(K)(Cl)	−0.49	0.36	2	−212
22	(Mg)(Cl)	0.15	0.66	4	212
23	(Ca)(Cl)	0.00	0.66	4	212
24	(NH_4_)(Cl)	−0.51	0.36	2	−212
25	(SO_4_)(H)	1.79	0.89	4	1166
26	(Na)(SO_4_)	0.39	0.99	4	53
27	(K)(SO_4_)	0.52	0.89	4	217
28	(Mg)(SO_4_)	1.65	1.80	8	307
29	(Ca)(SO_4_)	1.60	1.76	8	376
30	(NH_4_)(SO_4_)	0.92	0.65	4	53
31	(PO_4_)(H)	11.65	0.96	6	−1484
32	(PO_4_)(H)_2_	18.48	1.61	10	−1675
33	(PO_4_)(H)_3_	20.06	1.97	12	−1299
34	(Na)(PO_4_)	0.96	0.96	6	371
35	(Na)(PO_4_)(H)	12.42	1.61	10	−901
36	(Na)(PO_4_)(H)_2_	18.70	1.97	12	−742
37	(Na)_2_(PO_4_)	1.76	1.61	10	424
38	(Na)_2_(PO_4_)(H)	12.14	1.97	12	−848
39	(K)(PO_4_)	0.86	0.96	6	318
40	(K)(PO_4_)(H)	12.23	1.61	10	−742
41	(K)(PO_4_)(H)_2_	18.50	1.97	12	−1378
42	(K)_2_(PO_4_)	1.40	1.61	10	371
43	(K)_2_(PO_4_)(H)	12.17	1.97	12	−689
44	(Ca)(PO_4_)(H)	13.59	2.21	14	−848
45	(Ca)(PO_4_)(H)_2_	19.55	2.27	14	−1039
46	(Mg)(PO_4_)(H)	13.73	2.21	14	−848
47	(Mg)(PO_4_)(H)_2_	19.68	2.27	14	−1039
48	(NH_4_)(PO_4_)	0.96	0.96	6	371
49	(NH_4_)(PO_4_)(H)	12.48	1.34	10	−901
50	(NH_4_)(PO_4_)(H)_2_	18.70	1.97	12	−742
51	(NH_4_)_2_(PO_4_)	1.76	1.61	10	371
52	(NH_4_)_2_(PO_4_)(H)	12.14	1.97	12	−689
53	(Na)(K)(PO_4_)	1.94	1.61	10	398
54	(Na)(NH_4_)(PO_4_)	2.11	1.61	10	398
55	(K)(NH_4_)(PO_4_)	1.94	1.61	10	398
56	(Na)(K)(PO_4_)(H)	12.46	1.97	12	716
57	(Na)(NH_4_)(PO_4_)(H)	12.44	1.97	12	716
58	(K)(NH_4_)(PO_4_)(H)	12.46	1.97	12	716
59	(AA)(H)	9.28	0.57	2	−2325
60	(AA)(H)_2_	11.62	0.65	2	−143
61	(Na)(AA)	−0.68	0.64	2	0
62	(Na)(AA)(H)	8.74	0.69	2	−2325
63	(K)(AA)	−0.68	0.64	2	53
64	(K)(AA)(H)	8.74	0.69	2	−2325
65	(NH_4_)(AA)	−0.68	0.64	2	53
66	(NH_4_)(AA)(H)	8.74	0.69	2	−2325
67	(Mg)(AA)	1.67	0.97	4	1325
68	(Mg)(AA)(H)	9.74	0.54	2	1000
69	(Ca)(AA)	0.97	0.92	4	−212
70	(Ca)(AA)(H)	9.75	0.79	2	424
71	(SO_4_)(AA)(H)	10.28	0.78	2	−2219
72	(Cl)(AA)(H)	10.98	1.20	2	−2272
73	(F)(AA)(H)	11.18	1.20	2	−2219
74	(SCN)(AA)(H)	10.98	1.20	2	−2272
75	(CO_3_)(H)	9.85	0.66	4	−774
76	(CO_3_)(H)_2_	15.97	1.01	6	−1261
77	(Na)(CO_3_)	0.80	0.66	4	53
78	(Na)(CO_3_)(H)	9.87	1.01	6	−721
79	(K)(CO_3_)	0.61	0.66	4	217
80	(K)(CO_3_)(H)	9.79	1.01	6	−557
81	(Ca)(CO_3_)	2.56	1.26	8	795
82	(Ca)(CO_3_)(H)	10.86	1.31	8	233
83	(Mg)(CO_3_)	2.22	1.26	8	530
84	(Mg)(CO_3_)(H)	10.56	1.31	8	−509
85	(NH_4_)(CO_3_)	0.80	0.66	4	53
86	(NH_4_)(CO_3_)(H)	9.87	1.01	6	−721
87	(Urea)(H)	0.14	0.06	0	−212
88	(Urea)_2_(H)	−0.57	0.11	0	−159
89	(Ca)(Urea)	−0.80	0.06	0	53
90	(Mg)(Urea)	−0.30	0.06	0	53
91	(SO_4_)(Urea)(H)	1.11	0.71	4	1166
92	(PO_4_)(Urea)(H)_2_	17.48	1.67	10	−1675
93	(PO_4_)(Urea)(H)_3_	20.18	2.02	12	−1299

^(a)^Charges omitted for simplicity; ^(b)^±0.01 − 0.1 standard deviation.

**Table 4:** Stability constants of SALMO species at *t* = 25, 30, 37, and 40°C and *I*_ref_ ionic strength (by (11)).

ID	Species^(a)^	log⁡*β*_ref_^(b)^			
		*t* = 25°C	*t* = 30°C	*t* = 37°C	*t* = 40°C
1	(OH)	−13.82	−13.66	−13.45	−13.36
2	(Na)(OH)	−13.59	−13.45	−13.25	−13.17
3	(K)(OH)	−13.65	−13.53	−13.35	−13.28
4	(Ca)(OH)	−12.87	−12.74	−12.56	−12.49
5	(Mg)(OH)	−11.73	−11.61	−11.45	−11.38
6	(NH_4_)(OH)	−9.26	−9.10	−8.90	−8.82
7	(SCN)(H)	−1.66	−1.51	−1.29	−1.20
8	(Na)(SCN)	−0.52	−0.52	−0.51	−0.51
9	(K)(SCN)	−0.50	−0.50	−0.49	−0.49
10	(Mg)(SCN)	−1.20	−1.19	−1.19	−1.19
11	(Ca)(SCN)	−1.20	−1.19	−1.19	−1.19
12	(NH_4_)(SCN)	−0.52	−0.52	−0.51	−0.51
13	(F)(H)	2.96	2.99	3.05	3.07
14	(F)_2_(H)	3.52	3.53	3.55	3.56
15	(Na)(F)	−0.60	−0.56	−0.51	−0.49
16	(K)(F)	−0.58	−0.54	−0.49	−0.47
17	(Mg)(F)	1.28	1.31	1.36	1.39
18	(Ca)(F)	0.67	0.71	0.76	0.79
19	(NH_4_)(F)	−0.60	−0.56	−0.51	−0.49
20	(Na)(Cl)	−0.46	−0.48	−0.51	−0.53
21	(K)(Cl)	−0.46	−0.48	−0.49	−0.50
22	(Mg)(Cl)	0.12	0.14	0.15	0.16
23	(Ca)(Cl)	−0.03	−0.02	0.00	0.01
24	(NH_4_)(Cl)	−0.48	−0.50	−0.51	−0.52
25	(SO_4_)(H)	1.64	1.71	1.79	1.83
26	(Na)(SO_4_)	0.38	0.38	0.39	0.39
27	(K)(SO_4_)	0.50	0.51	0.52	0.53
28	(Mg)(SO_4_)	1.61	1.63	1.65	1.66
29	(Ca)(SO_4_)	1.55	1.57	1.60	1.61
30	(NH_4_)(SO_4_)	0.91	0.92	0.92	0.92
31	(PO_4_)(H)	11.84	11.76	11.65	11.60
32	(PO_4_)(H)_2_	18.70	18.60	18.48	18.43
33	(PO_4_)(H)_3_	20.23	20.16	20.06	20.02
34	(Na)(PO_4_)	0.91	0.93	0.96	0.97
35	(Na)(PO_4_)(H)	12.54	12.49	12.42	12.39
36	(Na)(PO_4_)(H)_2_	18.80	18.76	18.70	18.68
37	(Na)_2_(PO_4_)	1.70	1.73	1.76	1.77
38	(Na)_2_(PO_4_)(H)	12.25	12.20	12.14	12.11
39	(K)(PO_4_)	0.81	0.83	0.86	0.87
40	(K)(PO_4_)(H)	12.33	12.28	12.23	12.21
41	(K)(PO_4_)(H)_2_	18.68	18.60	18.50	18.46
42	(K)_2_(PO_4_)	1.35	1.37	1.40	1.41
43	(K)_2_(PO_4_)(H)	12.26	12.22	12.17	12.15
44	(Ca)(PO_4_)(H)	13.70	13.66	13.59	13.57
45	(Ca)(PO_4_)(H)_2_	19.69	19.63	19.55	19.52
46	(Mg)(PO_4_)(H)	13.84	13.80	13.73	13.71
47	(Mg)(PO_4_)(H)_2_	19.82	19.76	19.68	19.65
48	(NH_4_)(PO_4_)	0.91	0.93	0.96	0.97
49	(NH_4_)(PO_4_)(H)	12.60	12.55	12.48	12.45
50	(NH_4_)(PO_4_)(H)_2_	18.80	18.76	18.70	18.68
51	(NH_4_)_2_(PO_4_)	1.71	1.73	1.76	1.77
52	(NH_4_)_2_(PO_4_)(H)	12.23	12.19	12.14	12.12
53	(Na)(K)(PO_4_)	1.89	1.91	1.94	1.95
54	(Na)(NH_4_)(PO_4_)	2.06	2.08	2.11	2.12
55	(K)(NH_4_)(PO_4_)	1.89	1.91	1.94	1.95
56	(Na)(K)(PO_4_)(H)	12.37	12.41	12.46	12.48
57	(Na)(NH_4_)(PO_4_)(H)	12.35	12.39	12.44	12.46
58	(K)(NH_4_)(PO_4_)(H)	12.37	12.41	12.46	12.48
59	(AA)(H)	9.58	9.45	9.28	9.21
60	(AA)(H)_2_	11.64	11.63	11.62	11.61
61	(Na)(AA)	−0.68	−0.68	−0.68	−0.68
62	(Na)(AA)(H)	9.04	8.91	8.74	8.67
63	(K)(AA)	−0.68	−0.68	−0.68	−0.67
64	(K)(AA)(H)	9.04	8.91	8.74	8.67
65	(NH_4_)(AA)	−0.68	−0.68	−0.68	−0.67
66	(NH_4_)(AA)(H)	9.04	8.91	8.74	8.67
67	(Mg)(AA)	1.49	1.57	1.67	1.71
68	(Mg)(AA)(H)	9.61	9.66	9.74	9.77
69	(Ca)(AA)	1.00	0.99	0.97	0.97
70	(Ca)(AA)(H)	9.70	9.72	9.75	9.76
71	(SO_4_)(AA)(H)	10.56	10.44	10.28	10.21
72	(Cl)(AA)(H)	11.27	11.15	10.98	10.91
73	(F)(AA)(H)	11.47	11.34	11.18	11.11
74	(SCN)(AA)(H)	11.27	11.15	10.98	10.91
75	(CO_3_)(H)	9.95	9.91	9.85	9.83
76	(CO_3_)(H)_2_	16.13	16.06	15.97	15.93
77	(Na)(CO_3_)	0.79	0.80	0.80	0.80
78	(Na)(CO_3_)(H)	9.96	9.92	9.87	9.85
79	(K)(CO_3_)	0.58	0.59	0.61	0.62
80	(K)(CO_3_)(H)	9.86	9.83	9.79	9.77
81	(Ca)(CO_3_)	2.46	2.50	2.56	2.58
82	(Ca)(CO_3_)(H)	10.83	10.84	10.86	10.87
83	(Mg)(CO_3_)	2.15	2.18	2.22	2.24
84	(Mg)(CO_3_)(H)	10.63	10.60	10.56	10.54
85	(NH_4_)(CO_3_)	0.79	0.80	0.80	0.80
86	(NH_4_)(CO_3_)(H)	9.96	9.92	9.87	9.85
87	(Urea)(H)	0.17	0.16	0.14	0.13
88	(Urea)_2_(H)	−0.55	−0.56	−0.57	−0.57
89	(Ca)(Urea)	−0.81	−0.80	−0.80	−0.80
90	(Mg)(Urea)	−0.31	−0.30	−0.30	−0.30
91	(SO_4_)(Urea)(H)	0.96	1.02	1.11	1.15
92	(PO_4_)(Urea)(H)_2_	17.69	17.60	17.48	17.43
93	(PO_4_)(Urea)(H)_3_	20.35	20.28	20.18	20.14

^(a)^Charges omitted for simplicity; ^(b)^±0.01 − 0.1 standard deviation.

**Table 5:** Free concentrations of various components of synthetic saliva at *t* = 25 and 37°C, at pH = 6.5 and 7.0.

Component (X)	[X]/mol L^−1^		pX		%[X]^(a)^	
	*t* = 25°C					
	pH = 6.5	pH = 7.0	pH = 6.5	pH = 7.0	pH = 6.5	pH = 7.0
Ca^2+^	1.499*E* − 03	1.322*E* − 03	2.824	2.879	71.4 ± 1.4	63.0 ± 1.8
Mg^2+^	3.243*E* − 04	2.757*E* − 04	3.489	3.560	64.9 ± 1.8	55.1 ± 2.2
Na^+^	1.952*E* − 02	1.934*E* − 02	1.709	1.713	96.2 ± 0.5	95.3 ± 0.5
K^+^	2.800*E* − 02	2.784*E* − 02	1.553	1.555	96.9 ± 0.4	96.3 ± 0.4
NH_4_^+^	3.347*E* − 03	3.292*E* − 03	2.475	2.483	95.6 ± 0.4	94.1 ± 0.5
SCN^−^	1.903*E* − 03	1.903*E* − 03	2.720	2.720	97.6 ± 0.6	97.6 ± 0.6
F^−^	2.401*E* − 06	2.406*E* − 06	5.620	5.619	96.0 ± 0.7	96.2 ± 0.6
Cl^−^	2.460*E* − 02	2.461*E* − 02	1.609	1.609	97.2 ± 0.6	97.3 ± 0.6
SO_4_^2−^	8.604*E* − 04	8.657*E* − 04	3.065	3.063	78.2 ± 1.0	78.7 ± 1.0
HPO_4_^2−^	1.954*E* − 03	3.594*E* − 03	2.709	2.444	23.0 ± 0.7	42.3 ± 0.8
AA	1.288*E* − 07	4.058*E* − 07	6.890	6.392	0.0 ± 0.1	0.1 ± 0.1
HCO_3_^−^	7.166*E* − 03	9.270*E* − 03	2.145	2.033	62.6 ± 1.0	81.0 ± 0.6
Urea	3.297*E* − 03	3.298*E* − 03	2.482	2.482	99.9 ± 0.1	99.9 ± 0.1

	*t* = 37°C					
	pH = 6.5	pH = 7.0	pH = 6.5	pH = 7.0	pH = 6.5	pH = 7.0

Ca^2+^	1.413*E* − 03	1.231*E* − 03	2.850	2.910	67.3 ± 1.5	58.6 ± 1.9
Mg^2+^	3.161*E* − 04	2.686*E* − 04	3.500	3.571	63.2 ± 1.8	53.7 ± 2.2
Na^+^	1.946*E* − 02	1.928*E* − 02	1.711	1.715	95.9 ± 0.4	95.0 ± 0.5
K^+^	2.794*E* − 02	2.775*E* − 02	1.554	1.557	96.7 ± 0.4	96.0 ± 0.4
NH_4_^+^	3.327*E* − 03	3.251*E* − 03	2.478	2.488	95.1 ± 0.4	92.9 ± 0.5
SCN^−^	1.904*E* − 03	1.904*E* − 03	2.720	2.720	97.6 ± 0.6	97.6 ± 0.6
F^−^	2.387*E* − 06	2.393*E* − 06	5.622	5.621	95.5 ± 0.8	95.7 ± 0.7
Cl^−^	2.465*E* − 02	2.465*E* − 02	1.608	1.608	97.4 ± 0.5	97.4 ± 0.5
SO_4_^2−^	8.513*E* − 04	8.575*E* − 04	3.070	3.067	77.4 ± 1.0	78.0 ± 1.0
HPO_4_^2−^	1.966*E* − 03	3.541*E* − 03	2.706	2.451	23.1 ± 0.7	41.7 ± 0.8
AA	2.563*E* − 07	8.072*E* − 07	6.591	6.093	0.1 ± 0.1	0.2 ± 0.0
HCO_3_^−^	7.509*E* − 03	9.417*E* − 03	2.124	2.026	65.6 ± 0.9	82.2 ± 0.5
Urea	3.297*E* − 03	3.298*E* − 03	2.482	2.482	99.9 ± 0.1	99.9 ± 0.1

^(a)^±95% confidence interval on the formation percentage.

**Table 6:** Main parameters of the Single Saliva Salt (MX) for the inorganic components of synthetic saliva, according to SALMO model.

Characteristic	Symbol	Value	Unit
Salt concentration	*c*_MX_	0.0518	mol L^−1^
Ionic strength	*I*_MX_	0.0701	mol L^−1^
Charge	*z*	±1.163	—
Salinity	*S*	4.37	(‰)

**Table 7:** Stability constants of S_3_M species at *t* = 37°C and *I*_ref_ ionic strength and corresponding parameters for their dependence on ionic strength and temperature (by (8)–(11)).

Equilibrium	log⁡*β*_ref_^(a)^	*I*_ref_	*C*	*z* ^*^	*a*
H^+^ + X^1.163−^ = HX^0.163−^	3.02 ± 0.04	0.05	2.326	0.404	−77
X^1.163−^ = H_−1_X^2.163−^ + H^+^	−13.45 ± 0.05	0.05	−4.326	−0.704	0
M^1.163^ + X^1.163−^ = MX	0.48 ± 0.02	0.05	2.705	0.461	154
M^1.163^ = M(OH)^0.163^ + H^+^	−13.15 ± 0.02	0.05	0.326	−0.006	1926

^(a)^±standard deviation.
